# Genetic Analysis of Iranian Patients with Familial Hypercholesterolemia

**DOI:** 10.22034/ibj.22.2.117

**Published:** 2018-03

**Authors:** Mahdis Ekrami, Maryam Torabi, Soudeh Ghafouri-Fard, Javad Mowla, Bahram Mohammad Soltani, Feyzollah Hashemi-Gorji, Zahra Mohebbi, Mohammad Miryounesi

**Affiliations:** 1 Department of Genetics, Faculty of Biological Sciences, Tarbiat Modares University, Tehran, Iran; 2 Department of Genetics, Faculty of Basic Sciences, Islamic Azad University, Zanjan Branch, Zanjan, Iran; 3 Department of Medical Genetics, School of Medicine, Shahid Beheshti University of Medical Sciences, Tehran, Iran; 4 Genomic Research Center, Shahid Beheshti University of Medical Sciences, Tehran, Iran

**Keywords:** Apolipoprotein B 100, Hypercholesterolemia, Genetics, Low-density lipoprotein receptor

## Abstract

**Background:**

Familial hypercholesterolemia (FH) is a frequent autosomal dominant disorder of lipoprotein metabolism. This disorder is generally caused by mutations in low-density lipoprotein receptor (*LDLR*), apolipoprotein B 100 (*APOB*), and proprotein convertase subtilisin/kexin type 9 (*PCSK9*) genes. In the present study, we aimed at identifying the common *LDLR* and *APOB* gene mutations in an Iranian population.

**Methods:**

Eighty unrelated Iranian patients with FH entered the study, based on Simon Broome diagnostic criteria. All samples were screened for two common *APOB* gene mutations, including R3500Q and R3500W, by the means of ARMS-PCR and PCR- RFLP assays, respectively. In addition, exons 3, 4, 9, and 10 of *LDLR* gene were sequenced in all patients.

**Results:**

A novel mutation in exon 3 (C95W) and a previously described mutation in exon 4 (D139H) of *LDLR* gene were found. Three previously reported polymorphisms in *LDLR* gene as well as three novel polymorphisms were detected in the patients. However, in the studied population, no common mutations were observed in *APOB* gene.

**Conclusion:**

The results of our study imply that the genetic basis of FH in Iranian patients is different from other populations.

## INTRODUCTION

Familial hypercholesterolemia (FH; MIM# 143890) is a frequent autosomal dominant disorder of lipoprotein metabolism distinguished by an increase in LDL-C, tendon xanthomata, arcus cornea, and the high risk of coronary heart disease (CHD)[1]. In heterozygous FH patients, a twofold to threefold increase in LDL-C is observed, which leads to CHD before age 45, if untreated. Homozygous FH patients show a more severe phenotype distinguished by sixfold to eightfold increase in plasma LDL-C and CHD before the age of 12.5 years in untreated patients[2]. To decrease the morbidity and mortality of this disorder, it is necessary to diagnose and treat high-risk patients. Although patients are typically diagnosed by their elevated LDL-C levels, clinical features, and familial history, the genetic testing is regarded as a conclusive approach in diagnosis of FH through the identification of pathological mutations. Subsequently, first-degree relatives are subjected to cascade testing by using lipid level measurments and genetic tests. Such strategy has been suggested as the best strategy, due to its cost-effectiveness[3]. The main genetic causes of this disorder are mutations in the gene encoding the low-density lipoprotein receptor (*LDLR*). This cell-surface receptor is responsible for LDL-C uptake by endocytosis and its subsequent degradation in liver.

*LDLR* gene is located on chromosome 19p13.3 and contains 18 exons. A wide range of mutations such as insertions, deletions, nonsense, and missense mutations has been identified in FH patients so far[1].

The step-wise strategy for identification of mutations starts with testing for some common mutations and searching for large deletions or re-arrangements in the *LDLR* gene. Afterwards, the entire coding and control regions of this gene is screened by direct sequencing or by the methods called fluorescent single-strand conformation polymorphism test (SSCP) and denaturing high-performance liquid chromatography test (dHPLC). The mentioned strategy is expected to detect the cause of FH in a substantial proportion of people[4]. Furthermore, mutations in apolipoprotein B 100 (*APOB*) and proprotein convertase subtilisin/kexin type 9 (*PCSK9*) genes have been associated with FH[4]. In this study, we attempted to identify two common *APOB* gene mutations (R3500Q and R3500W) as well as *LDLR* gene mutations in a population of 80 Iranian patients with definite or possible FH.

## MATERIALS AND METHODS

### Patients

In total, 80 unrelated patients with definite or possible FH were enrolled in the study according to the Simon Broome diagnostic criteria[4]. Patients (55 males and 25 females) were selected from suspected FH patients referred to Genomic Research Center in Tehran (Iran) during 2016. Adult patients with total cholesterol levels > 290 mg/dL (7.5 mmol/L) or LDL-C >190 mg/dL (4.9 mmol/L) and children less than 16 years with total cholesterol levels >260 mg/dL (6.7 mmol/L) or LDL-C >155 mg/dL (4.0 mmol/L) were regarded to have definite FH, if tendon xanthomas were detected in them or in their first- or second-degree relative. These patients were regarded as possible FH if they had the family history of myocardial infarction at age 60 or younger in first-degree relative or age 50 years or younger in second-degree relative or the family history of elevated total cholesterol in first- or second-degree relative. Demographic and laboratory parameters of all patients obtained through questionnaires and interviews are summarized in Table 1. Patients with diabetes mellitus, hypertension, renal disease, hypothyroidism, and steroid therapy were excluded from the study. All patients aged between 9 to 67 years. Informed consents were obtained from all patients before collecting blood samples. Glucose, total cholesterol, triglycerides, HDL-C, and LDL-C were assayed using standard protocols. The study protocol was approved by the local ethical committee.

**Table 1:** Demographic and laboratory parameters of all patients

Parameters	Mean ± SD
Age (year)	48.2 ± 17.5
BMI (Kg/m^2^)	23.35 ± 3.2
Systolic blood pressure (mmHg)	122.32 ± 7.3
Diastolic blood pressure (mmHg)	88.7 ± 2.4
Total Cholesterol (mg/dl)	356 ± 24.6
Trigyceride (mg/dl)	108.2 ± 9.2
HDL-C (mg/dl)	54.0 ± 2.1
LDL-C (mg/dl)	182.0 ± 5.6
VLDL-C (mg/dl)	22.0 ± 1.3

### Genetic study

Genomic DNA was extracted from peripheral blood using standard salting-out method. The primer sequences, annealing temperatures, and restriction enzyme used for each reaction are shown in Table 2. PCR was carried out in a thermal cycler system (Applied Biosystems, CA, USA) using a commercial Master mix (Kawsar Biotech Company, Iran). The PCR program included a primary denaturation at 95ºC for 5 minutes, followed by 35 cycles of 95ºC for 45 seconds, specific annealing temperatures for 35 seconds, and 72ºC for 60 seconds, with the final extension of 72ºC for 5 minutes. All *LDLR* PCR products were sequenced using an ABI 3100 DNA Sequencer (Applied Biosystems, CA, USA). For R3500W analysis, PCR products were then incubated with the restriction enzyme TaaI (HPYCH4III) (Fermentas, Lithuania) at 65 °C for 16 h. If C nucleotide exists at the polymorphic site, this enzyme will cut 267-bp PCR products into two 163 and 104 fragments. If T nucleotide exists, PCR product remains intact.

**Table 2:** The primer sequences, annealing temperatures, and restriction enzyme used for each reaction

Gene	Sequence (5’→3’)	Product size (bp)	Annealing temperature (°C)	Restriction enzyme
*LDLR*				
Exon 3	F: CAGTGGGTCTTTCCTTTGAGTG	331	63	-
	R: GGGATTTGAAGGGCGGAAGAGG			
Exon 4	F: TGGGAAATGTGTACAGATGAGG	670	58	-
	R: ATCCACTTCGGCACCTAAATCA			
Exons 9-10	F: AGGATGACACAAGGGGATGGGG	726	63	-
	R: GTCAGGCTGGTCTTGATGATCC
*APOB*				
R3500Q	F: TTGAATTCCAAGAGCACACGGT	412	61	-
	R: TGTGCCTTTTCTTGGTCATTGGA			
R3500W	F: CTAAAGGAGCAGTTGACCACAAG	267	60.2	HpyCH4III
	R: GGGAATATATGCGTTGGAGTGTG			

## RESULTS

**Fig. 1:**
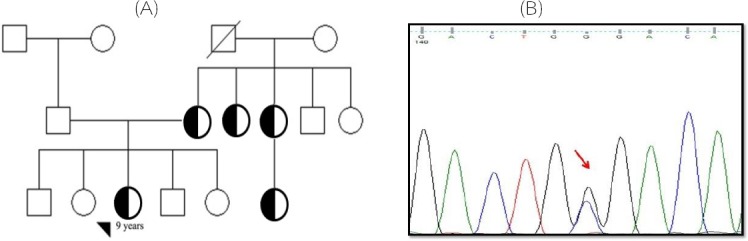
The patient’s pedigree with C95W mutation (c.285C>G) (A) and the detected nucleotide change (B). Arrow shows the mutation site

**Fig. 2:**
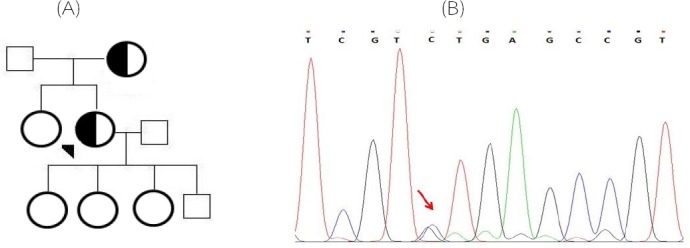
The patient’s pedigree with D139H (c.415G>C) mutation (A) and the detected nucleotide change (B). Arrow shows the mutation site

Totally, 80 FH individuals entered the study who met the Simon Broome Diagnostic criteria. All patients who were selected for further evaluations in the current study had the familial history of CHD. All samples were first analyzed for two *APOB* gene mutations, including R3500Q and R3500W. None of the patients showed these mutations. Screening for mutations in 4 exons of *LDLR* gene showed a novel mutation in exon 3 (C95W, c.285C>G) and a previously described mutation in exon 4 (D139H, c.415G>C). Three previously reported polymorphisms (rs5930A/G, rs1003723C/T, and rs1569372A/G) as well as three novel intronic polymorphisms (c.313-97 G>A, c.313-64 A>T, and c.313 + 70 G>C) were detected in the patients. Table 3 shows the allele and genotype frequencies for the mentioned polymorphisms. The patient carrying C95W mutation was a nine-year-old female with a history of CHD and tendinous xanthomas. Figure 1 shows the patient’s pedigree and the detected mutation. The D139H mutation was found in a 65-year-old female patient who had both CHD and tendinous xanthomas. Figure 2 demonstrates her pedigree and the detected mutation.

**Table 3:** The allele and genotype frequencies for the *LDLR* polymorphisms

SNP	chr19: 11224181C>T	chr19: 11224265A>G	chr19: 11224491A>G
	rs1003723 (%)	rs5930 (%)	rs1569372 (%)
Genotypes	CC (53.4)	GG (37.2)	GG (7)
	CT (34.8)	AG (44.1)	AG (53)
	TT (11.8)	AA (18.6)	AA (40)
Alleles	C (61.7)	A (60)	A (55)
	T (38.3)	G (40)	G (45)

## DISCUSSION

In the present study, we aimed at identifying the common *LDLR* and *APOB* gene mutations in an Iranian population. Previously, it has been revealed that 93% of detected mutations in FH patients are located in *LDLR* gene, whereas the *APOB* variant (R3500Q) and *PCSK9* mutation account for approximately 5% and 1.7% of FH patients[5]. Therefore, in the current study, we did not include

*CSK9* mutation screening. The increased serum cholesterol levels that is typical for heterozygous FH result in a more than 50% risk of CHD by the age of 50 years in male patients and at least 30% in female by the age of 60 years.

Homozygous FH is infrequent and is associated with early demise from CHD[4]. Because of overlapping affected and unaffected individuals at LDL-C levels, the use of FH-specific cut-offs for LDL-C levels leads to diagnostic uncertainty in about 15% of children (aged 5-15 years) and in approximately half of the adults aged 45-55 years[2]. Consequently, identification of disease-causing mutations in each family is of practical importance. For FH, the suggested cascade testing is performed by the measurement of LDL-C in the blood and/or a DNA test, if a disease-causing mutation has been detected in the index case[4]. Due to the large size of coding region for *LDLR* gene, the identification of mutation hot spots in this gene is desirable. A study that enrolled more than 200 FH patients from the United Kingdom has revealed that the highest numbers of *LDLR* mutations were located in exons 3 (10%), 4 (28%), 10 (10%), and 14 (21%); 46% of *LDLR* mutations were detected in the ligand-binding domain (exons 3 to 6), and 46% were detected in the epidermal growth factor precursor-like domain (exons 7 to 14)[6]. Another study in Indian patients has shown exons 3 and 4 mutations of *LDLR* gene in 42 classical cases of FH with high LDL-C, xanthelasmas, tendon xanthomas, and arcus cornea[7]. Mutation screening of the *LDLR* gene in seven South African Indian patients with FH has indicated abnormal mobility heteroduplex and/or SSCP bands in exons 4, 9, and 16 of the *LDLR* gene in five of them[8]. In a study in 35 unrelated FH patients from Brazilian population, mutations have been found in 22 individuals. However, the distribution of mutations in these exons was different when tryptophan is present at this position from previously reported mutation spectrum in the United Kingdom[9]. Another study attempted to identify *LDLR* mutations in a group of 154 unrelated FH patients from a northern area of Malaysia by denaturing high-performance liquid chromatography. Based on the result, 29 gene sequence variants including eight different mutations in patients were detected[10]. There are at least three studies in Iranian FH patients. Fard-Esfahani *et al*.[11,12] reported a new missense mutation (445G>T) and a new frameshift mutation (660-661InsCC) in exon 4 in a total of about 30 Iranian FH patients. Farrokhi and co-workers[1] screened *LDLR* mutations throughout the entire coding region and reported no mutations in 30 unrelated patients with possible FH[1]. The technique used in the mentioned study was PCR-SSCP and subsequent sequencing of fragments with shifted pattern. However, in the current study, we used direct sequencing of four exons, which previous studies suggested them as mutation hot spots in *LDLR* gene. The results of our study along with these three mentioned studies in Iranian population imply that the prevalence and/or distribution of *LDLR* mutations in Iranian population might be different from other populations. However, further studies with larger sample sizes are needed to evaluate mutations in entire coding region of *LDLR* gene to detect mutation spectrum. This would facilitate the diagnosis of at-risk patients and their relatives.

**Fig. 3:**
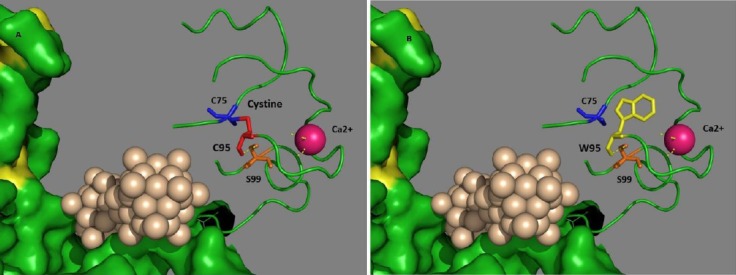
Three-dimensional structure of *LDLR* protein depicted by PyMol software. when Cysteine is present at position 95 (A) and

In the present study, we have identified a novel mutation (C95W) in exon 3 of *LDLR* gene in a nine-year-old female with a history of CHD and tendinous xanthomas. Although this novel variant has been found in the heterozygote state in the proband, the clinical manifestation of the proband is similar to the patients carrying homozygote mutations. Functional studies are required to explore if such variant results in a profound defect in the LDLR function. In addition, the presence of other variants within *LDLR* gene or other genes, which augment the effect of this mutation should not been ignored. Using Polyphen-2[13], *in silico* analysis predicted that this mutation is probably damaging with a score of 1 and revealed that this residue is conserved among all species. With this software, values nearer 1 are more assertively anticipated to be deleterious. Furthermore, Combined Annotation Dependent Depletion (CADD) tool, a software for analysis of the harmfulness of single nucleotide variants as well as insertion/deletions variants in the human genome[14], designated that this variant would be deleterious with a score of 23.0. In addition, PyMol software (the PyMOL Molecular Graphics System, Version 1.2r3pre, Schrödinger, LLC, USA) was used to make a three-dimensional structure of LDLR protein following C95W mutation (Fig. 3), which shows the aberrant protein configuration as a result of amino acid substitution which interfere with LDL binding to LDLR. It is worth noting that this mutation has occurred in the ligand binding domain. On the other hand, D139H mutation has previously been detected in a Malaysian FH patient and was predicted by PolyPhen-2 software to be probably damaging and was consequently considered as a pathogenic mutation[10]. The result of the current study provides further support for its pathogenicity.

In summary, eight *LDLR* gene sequence variants including two pathogenic ones were identified in the analyzed patients with a definite or possible diagnosis of FH. As the number of patients with identified mutations was low, and the whole coding region of the gene was not sequenced, it was not possible to compare LDL-C levels with patients with non-pathogenic variants. However, regarding the inconsistency with our results and the reports from other populations, it is necessary to evaluate mutation spectrum in our population. Such data would help in establishment of novel screening strategies in our population to identify individual gene defects in patients, which provides the possibility of early detection and the chance for CHD avoidance.
